# Targeting RACK1 to alleviate TDP-43 and FUS proteinopathy-mediated suppression of protein translation and neurodegeneration

**DOI:** 10.1186/s40478-023-01705-8

**Published:** 2023-12-18

**Authors:** Beibei Zhao, Catherine M. Cowan, Juliane A. Coutts, Darren D. Christy, Ananya Saraph, Shawn C. C. Hsueh, Stephen S. Plotkin, Ian R. Mackenzie, Johanne M. Kaplan, Neil R. Cashman

**Affiliations:** 1 University of British Columbia, Djavad Mowafaghian Centre for Brain Health, Vancouver, BC V6T 1Z3 Canada; 2 Department of Physics and Astronomy, University of British Columbia, Vancouver, BC V6T 1Z1 Canada; 3 ProMIS Neurosciences, Cambridge, MA 02142 USA

## Abstract

TAR DNA-binding protein 43 (TDP-43) and Fused in Sarcoma/Translocated in Sarcoma (FUS) are ribonucleoproteins associated with pathogenesis of amyotrophic lateral sclerosis (ALS) and frontotemporal lobar degeneration (FTLD). Under physiological conditions, TDP-43 and FUS are predominantly localized in the nucleus, where they participate in transcriptional regulation, RNA splicing and metabolism. In disease, however, they are typically mislocalized to the cytoplasm where they form aggregated inclusions. A number of shared cellular pathways have been identified that contribute to TDP-43 and FUS toxicity in neurodegeneration. In the present study, we report a novel pathogenic mechanism shared by these two proteins. We found that pathological FUS co-aggregates with a ribosomal protein, the Receptor for Activated C-Kinase 1 (RACK1), in the cytoplasm of spinal cord motor neurons of ALS, as previously reported for pathological TDP-43. In HEK293T cells transiently transfected with TDP-43 or FUS mutant lacking a functional nuclear localization signal (NLS; TDP-43^ΔNLS^ and FUS^ΔNLS^), cytoplasmic TDP-43 and FUS induced co-aggregation with endogenous RACK1. These co-aggregates sequestered the translational machinery through interaction with the polyribosome, accompanied by a significant reduction of global protein translation. RACK1 knockdown decreased cytoplasmic aggregation of TDP-43^ΔNLS^ or FUS^ΔNLS^ and alleviated associated global translational suppression. Surprisingly, RACK1 knockdown also led to partial nuclear localization of TDP-43^ΔNLS^ and FUS^ΔNLS^ in some transfected cells, despite the absence of NLS. In vivo, RACK1 knockdown alleviated retinal neuronal degeneration in transgenic *Drosophila melanogaster* expressing hTDP-43^WT^ or hTDP-43^Q331K^ and improved motor function of hTDP-43^WT^ flies, with no observed adverse effects on neuronal health in control knockdown flies. In conclusion, our results revealed a novel shared mechanism of pathogenesis for misfolded aggregates of TDP-43 and FUS mediated by interference with protein translation in a RACK1-dependent manner. We provide proof-of-concept evidence for targeting RACK1 as a potential therapeutic approach for TDP-43 or FUS proteinopathy associated with ALS and FTLD.

**Supplementary Information:**

The online version contains supplementary material available at 10.1186/s40478-023-01705-8.

## Introduction

The Receptor for Activated C-Kinase 1 (RACK1) is a member of the tryptophan, aspartic acid repeat (WD-repeat) protein family. It was first identified as a receptor for protein kinase C (PKC) βII to activate the translational activity of Eukaryotic Translation Initiation Factor 6 (eIF6), and regulates a variety of cellular events, such as cell growth, motility, differentiation, microRNA biogenesis and function, and protein synthesis [7, 8, 10, 21, 25, 33, 41, 42, 61, 66].

Similar to many other WD-repeat proteins, RACK1 is highly conserved throughout evolution from prokaryotes to eukaryotes [70], and adopts a seven-bladed β-propeller structure as confirmed by X-ray crystallography studies [13]. Also as with other WD-repeat proteins, RACK1 itself lacks catalytic enzymatic activities, but plays a crucial role in scaffolding numerous proteins, such as kinases, transmembrane receptors, ion channels, and ribosomal proteins that are involved in a wide range of cellular functions [1]. As such, RACK1 serves as a hub connecting a complex network of cellular signaling events.

To date, at least 80 proteins have been identified to interact with RACK1 [1], including the RACK1-ribosome association, which has drawn intense research interest in the past two decades since first identified by mass spectrometry [60]. It is now established that RACK1 is a key constituent of the eukaryotic small (40S) subunit of the ribosome and is located in the vicinity of the mRNA exit channel [60]. Association of RACK1 with the ribosome plays a crucial role in cap-dependent translation and the downstream signaling events involved [20, 45].

Given the diverse roles of RACK1 in cellular function mentioned above, it is not surprising that RACK1 has been implicated in many human diseases, such as cancer, developmental disorders, addiction, and cardiovascular diseases [1]. However, little is known about the role of RACK1 in the pathogenesis of neurodegenerative disorders, such as amyotrophic lateral sclerosis (ALS) and frontotemporal dementia (FTD).

ALS is the most common motor neuron disease characterized by the ultimately fatal progressive degeneration of motor neurons in the spinal cord and motor cortex of the brain, which leads to paralysis of muscles that control limb movement, speech, swallowing, and breathing. While only ~ 10% of ALS is familial and associated with various genetic mutations (fALS), the remaining ~ 90% is sporadic (sALS). Frontotemporal lobar degeneration (FTLD), a pathological process occurring in clinical syndrome FTD, is characterized by neuronal degeneration in the frontal and temporal lobes of the brain. The most common clinical presentations include abnormal behavior, language disorders, and disorders of movement. ALS and FTD are considered to belong to a common disease spectrum (ALS–FTD) because of overlaps in genetic, clinical symptoms, and pathologies [12], and ~ 20% of ALS patients fall into the ALS–FTD spectrum based on clinical criteria [47, 49, 56].

Among the pathological similarities between ALS and FTLD, one major hallmark is the accumulation of cytoplasmic inclusions of TAR DNA-binding protein 43 (TDP-43) or Fused in Sarcoma/Translocated in Sarcoma (FUS/TLS), which are both ubiquitously expressed in most cell types and tissues. TDP-43 proteinopathy occurs in 60–70% of fALS and 90–95% of sALS, whereas TDP-43 pathology is observed in 50% of FTD. FUS pathology accounts for 10% of fALS and 10% of FTD [38, 46]. FUS inclusions are associated only with mutations in ALS and wild-type FUS in a small percentage of FTLD with neuronal inclusions composed of an unidentified ubiquitinated protein (atypical FTLD-U; aFTLD-U) [43]. TDP-43 and FUS proteins share many similarities in their cellular functions, protein structures, and subcellular distribution. TDP-43 and FUS are both RNA-binding proteins that play a pivotal role in multiple aspects of RNA processing, such as alternative splicing, metabolism, transport, and transcriptional and translational regulation [29, 46, 55]. Structurally, TDP-43 and FUS are both heterogeneous nuclear ribonucleoproteins (hnRNPs) that possess RNA-recognition motifs (RRMs), glycine-rich domains, a prion-like low complexity domain (PrLCD), and a nuclear localization signal (NLS) [19, 36]. Under physiological conditions, TDP-43 and FUS shuttle between the nucleus and cytoplasm, with ~ 90% being localized to the nucleus at any given time [29, 46, 55]. Under pathological conditions, such as ALS and FTLD, TDP-43 or FUS can be depleted from the nucleus and mislocalized to the cytoplasm where they can form inclusions in affected neurons and glial cells.

To date, a number of mechanistic pathways have been identified to explain the pathogenic activities of TDP-43 and FUS in ALS and FTLD, including disruption of mitochondrial function, disturbance of proteostasis, pro-inflammatory responses, and oxidative stress [55]. Importantly, a recent study has reported that RACK1 co-aggregates with cytoplasmically mislocalized TDP-43 in both a transfected cell line and ALS spinal cord motor neurons [57]. In addition, this study shows that TDP-43 cytoplasmic aggregates significantly suppress global translation, a process that requires the association of TDP-43 with ribosome-bound RACK1 [57], suggesting a novel pathogenic mechanism underlying TDP-43-associated neurodegeneration through RACK1-mediated global translational suppression.

Considering the many similarities between TDP-43 and FUS, in the present study we sought to: (1) investigate whether TDP-43 and FUS may utilize a similar pathogenic pathway through co-aggregation with RACK1 and suppression of global translation; (2) better understand the molecular mechanism by which interaction of RACK1 with pathological TDP-43 or FUS alters the homeostasis of global translation; (3) explore an RNAi-based approach targeting RACK1 that could potentially ameliorate the adverse effects of pathogenic TDP-43 and FUS on cellular function and neurodegeneration in vitro and in vivo.

## Results

### Pathological TDP-43 and FUS co-aggregate with RACK1 in the cytoplasm of spinal cord motor neurons of ALS

**Fig. 1:**
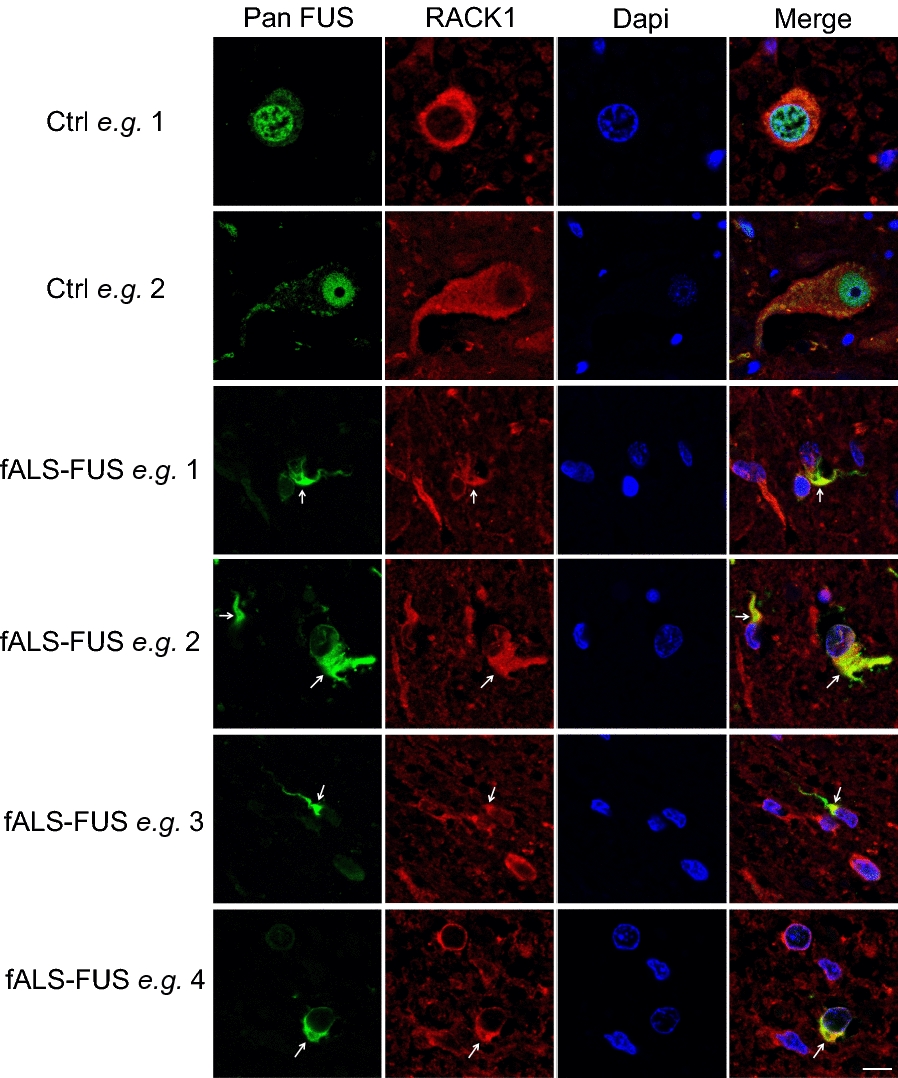
Immunohistochemical analysis shows co-aggregation of RACK1 with FUS in the cytoplasm (*arrows*) in spinal cord sections of a fALS-FUS-R521C case, in contrast to normal nuclear expression of FUS in control (Ctrl). Nuclei stained with DAPI in merged images. Scale bar: 10 μm

Using post-mortem human tissues, it has been shown that TDP-43 co-aggregates with RACK1 in the cytoplasm of spinal cord motor neurons from ALS spinal cords [57], a finding that we have confirmed (Additional file 1: Fig. S1). This led us to test whether pathological FUS may also co-aggregate with RACK1 in situ. Immunohistochemical analysis was performed on spinal cord sections from a fALS-FUS case carrying a R521C mutation. As shown in Fig. 1, in contrast to normal control spinal cords, where FUS and RACK1 were predominantly localized in the nucleus and cytoplasm, respectively, pathological FUS co-aggregated with RACK1 in the cytoplasm, similar to what was observed with TDP-43 [57].

### Cytoplasmic aggregates of nuclear localization signal-deficient mutants of TDP-43 and FUS are associated with RACK1 co-aggregation in HEK293T cells

**Fig. 2:**
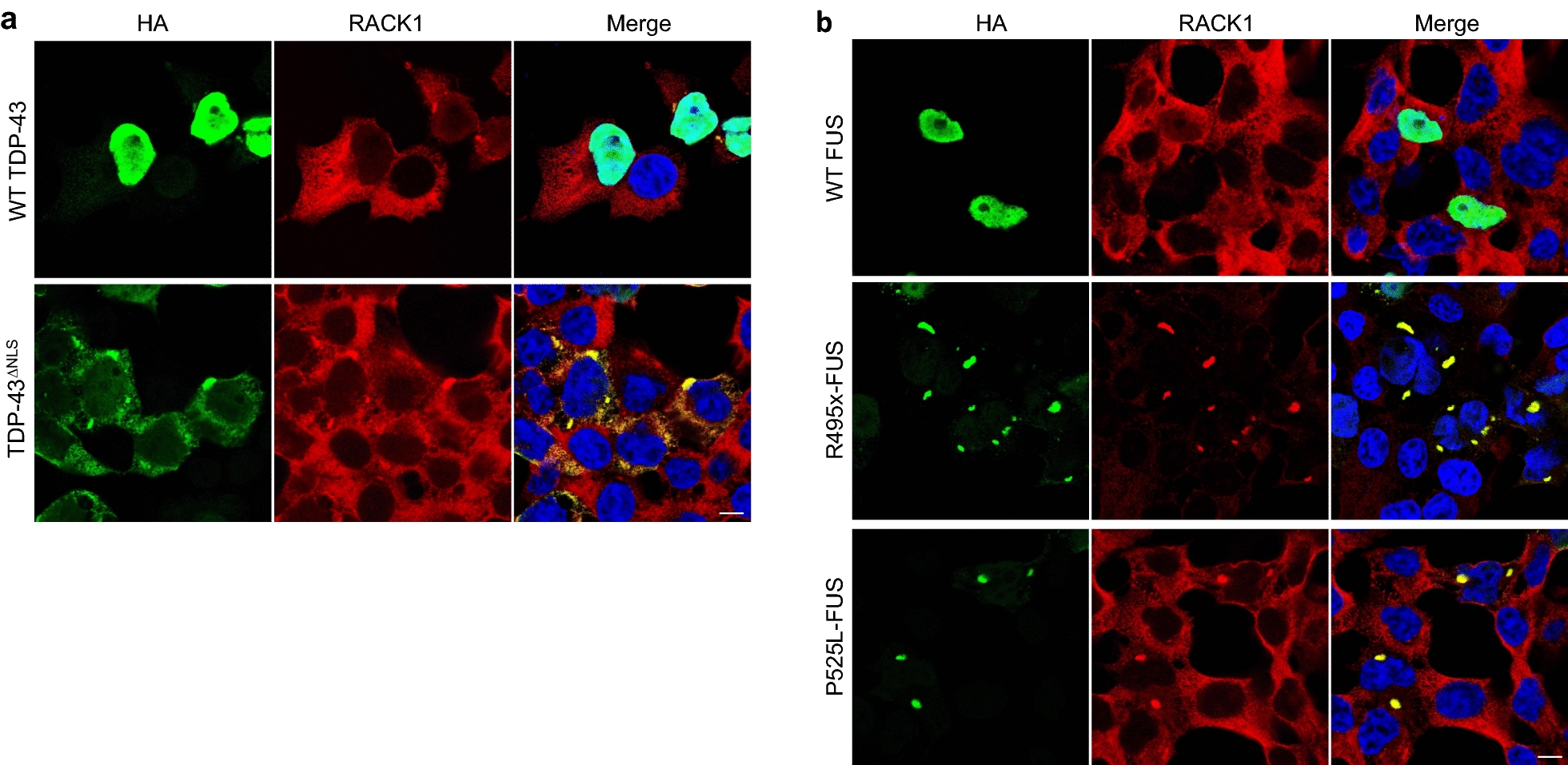
Immunocytochemical analysis demonstrates expression of HA-tagged TDP-43^ΔNLS^ (**a**, *bottom row*) or FUS^ΔNLS^ mutants, R495x- and P525L-FUS (**b**, *middle & bottom rows*), but not WT TDP-43 (**a**, *top row*) or WT-FUS (**b**, *top row*), which are primarily localized to the nucleus, is associated with co-aggregation of endogenous RACK1 in HEK293T cells. Nuclei stained with DAPI in merged images. Scale bars: 20 μm

To confirm and extend a previous finding that cytoplasmic TDP-43 co-aggregates with RACK1 in the neuroblastoma cell line SH-SY5Y [57], we transiently transfected HEK293T cells with HA-tagged wild-type (WT) TDP-43 or a nuclear localization signal (NLS)-deficient mutant of TDP-43, TDP-43^ΔNLS^. As shown in Fig. 2a, in WT TDP-43 over-expressing cells, where HA-WT TDP-43 was almost exclusively localized in the nucleus, endogenous RACK1 was diffusely expressed in the cytoplasm. In contrast, expression of the engineered mutant TDP-43^ΔNLS^, which aggregates in the cytoplasm, apparently induced RACK1 co-aggregation in the cytoplasm of HEK293T cells, consistent with the report in SH-SY5Y cells [57]. Similar experiments were performed using two variants of HA-tagged mutants of FUS that aggregate in the cytoplasm: natural fALS mutations R495x- and P525L-FUS, collectively referred to as FUS^ΔNLS^ herein. While RACK1 was diffusely expressed in the cytoplasm of WT FUS over-expressing cells, both FUS^ΔNLS^ mutants were associated with RACK1 co-aggregation in the cytoplasm (Fig. 2b). In contrast, expression of a ribosome binding deficient mutant of RACK1, R36D/K38E-RACK1 (DE-RACK1) [13], which forms large aggregates in both the cytoplasm and the nucleus, did not alter the normal nuclear distribution of endogenous TDP-43 and FUS (Additional file 1: Fig. S2), suggesting that it is TDP-43^ΔNLS^ and FUS^ΔNLS^ that played an initiating role in the formation of RACK1 co-aggregates and not the reverse. Transfection of HEK293T cells with a mutant disrupted in schizophrenia 1 (DISC1) with all tryptophan residues mutated to serines, 10W/S-DISC1, which forms large aggregates in the cytoplasm, displayed minimal co-aggregation with RACK1 (Additional file 1: Fig. S3), indicating that RACK1 aggregation is unlikely to be a generic consequence of cytoplasmic protein aggregation.

### RACK1 is misfolded when co-aggregated with pathological TDP-43 and FUS

**Fig. 3:**
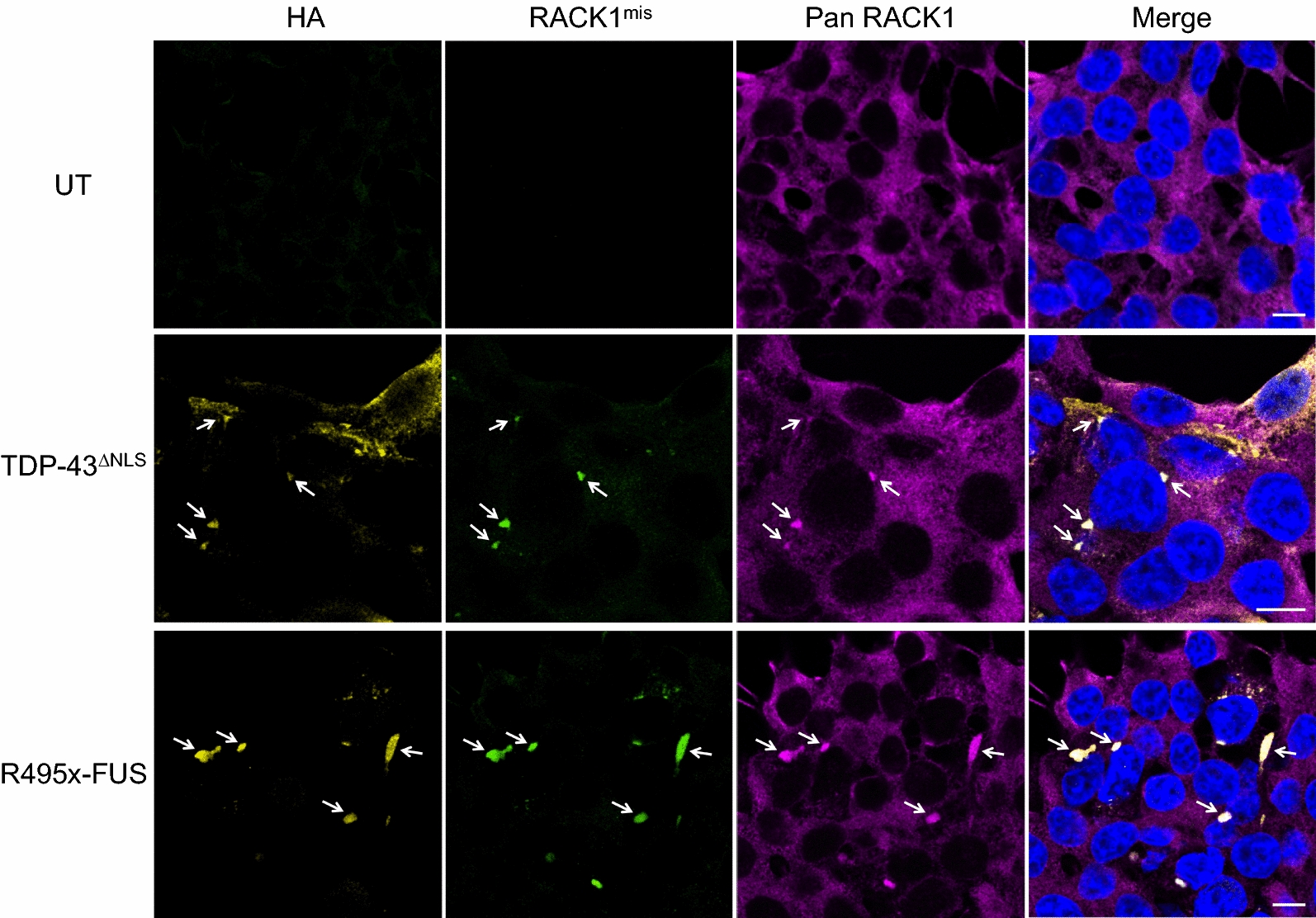
Immunocytochemical analysis demonstrates reactivity of RACK1 misfolding specific antibody “RACK1^mis^” with RACK1 in cytoplasmic aggregates of HA-tagged TDP-43^ΔNLS^ (*middle row*) or R495x-FUS (*bottom row*) transfected HEK293T cells but not with diffuse, non-aggregated RACK1 in the cytoplasm (stained by a Pan RACK1 antibody). RACK1^mis^ shows no reactivity with endogenous, physiological RACK1 in un-transfected (UT, *top row*) cells. Nuclei stained with DAPI in merged images. Scale bars: 10 μm

Using a computational modeling method we have developed, “Collective Coordinates” [48], we generated a rabbit monoclonal antibody, “RACK1^mis^”, against a misfolding-specific conformational epitope of RACK1. As shown in Fig. 3, RACK1^mis^ did not react with normal cytoplasmic RACK1 in un-transfected cells but specifically reacted with the species of RACK1 present in TDP-43^ΔNLS^ or FUS^ΔNLS^ co-aggregates, indicating that RACK1 is misfolded when co-aggregated with pathological TDP-43 or FUS. Furthermore, reactivity with misfolded RACK1 was also observed in post-mortem FTLD-TDP brain and sALS spinal cord tissue, but not in normal controls (Additional file 1: Fig. S4).

### Aggregates of TDP-43^ΔNLS^ and FUS^ΔNLS^ mutants suppress global translation

**Fig. 4:**
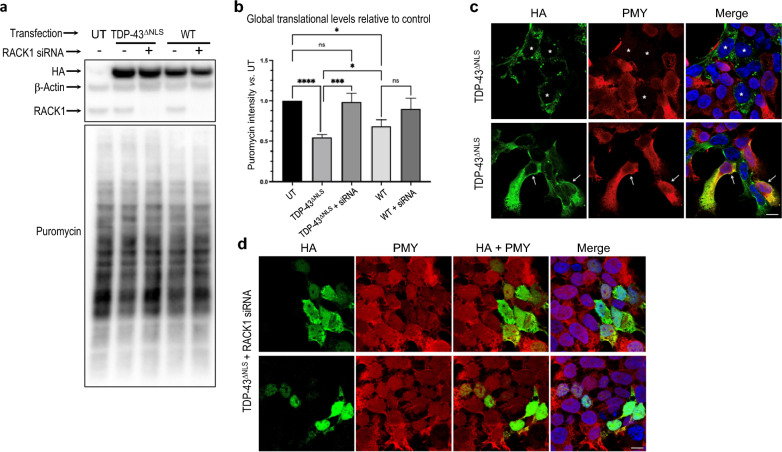
RACK1 knockdown alleviates aggregation and global translational suppression by TDP-43^ΔNLS^ and WT over-expression in HEK293T cells. **a** Representative SUnSET-Western Blot (WB) demonstrates that compared to control, expression of HA-tagged TDP-43^ΔNLS^ and to a lesser extent over-expression of WT TDP-43, induces a significant reduction in puromycin (PMY) incorporation. **b** RACK1 siRNA KD alleviates global translational levels in both cases as determined by quantification of PMY band intensities normalized to loading control β-actin. **c** SUnSET-ICC shows inhibition of PMY incorporation by TDP-43^ΔNLS^ (HA) expression preferably occurs in cells containing distinctive cytoplasmic aggregates (*asterisks, top row*). In cells where TDP-43^ΔNLS^ displays a filamentary expression pattern (*arrows, bottom row*), PMY incorporation is comparable to neighbouring HA negative UT cells. **d** RACK1 KD alleviates aggregation, resulting in diffuse cytoplasmic expression (*top row*) or predominantly nuclear localization (*bottom row*) of TDP-43^ΔNLS^ in a sub-population of transfected cells and correlating with normal PMY incorporation compared to neighbouring HA-negative UT cells. Nuclei stained with DAPI in merged images. *Statistics*: Ordinary one-way ANOVA Tukey multiple comparisons. n = 4 *p < 0.05; ***p < 0.001; ****p < 0.0001. Scale bars: 20 μm

**Fig. 5:**
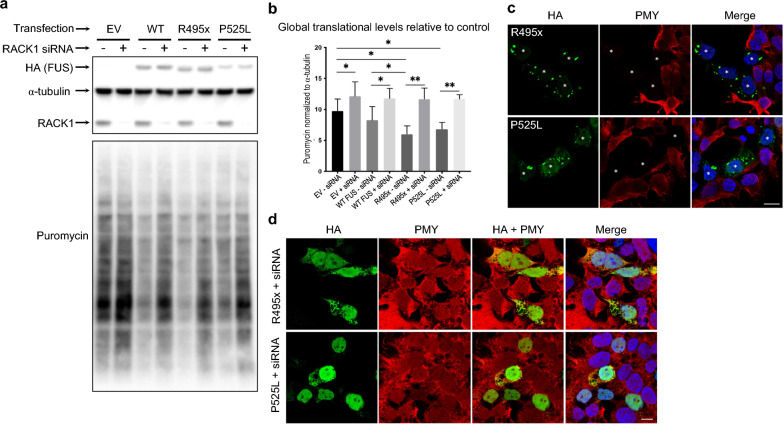
RACK1 knockdown alleviates aggregation and restores global translational suppression by FUS^ΔNLS^ mutants. **a** Representative SUnSET-Western Blot (WB) demonstrates that compared to control empty vector (EV) transfected cells, expression of HA-tagged FUS^ΔNLS^ mutants, R495x- and P525L-FUS, and to a lesser extent over-expression of WT FUS, induces a significant reduction in puromycin (PMY) incorporation. **b** siRNA KD alleviates global translational levels in all cases as determined by quantification of PMY band intensities normalized to loading control α-tubulin. **c** SUnSET-ICC demonstrates HA-tagged FUS^ΔNLS^ mutants, R495x- and P525L-FUS, cause aggregation and completely inhibit PMY incorporation in transfected cells (*asterisks*). **d** RACK1 KD alleviates aggregation, resulting in diffuse cytoplasmic expression or predominantly nuclear localization of HA-tagged FUS^ΔNLS^ mutants, R495x- and P525L-FUS, in a sub-population of transfected cells and correlating with normal PMY incorporation compared to neighbouring HA-negative UT cells. Nuclei stained with DAPI in merged images. *Statistics*: Student’s *t*-test, unpaired, two-tailed. *p < 0.05 and **p < 0.005. n = 5 Error bars: SEM. Scale bars: 20 μm

A body of evidence has suggested a role for RACK1 in translational regulation in either a ribosome-bound or a ribosome-free form [1, 45]. In particular, TDP-43^ΔNLS^ has been shown to suppress global translation through interaction with ribosome-bound RACK1 in neuroblastoma SH-SY5Y cells [57]. The in vitro and in situ results described above led us to hypothesize that the activities of RACK1-associated translational machinery may be similarly impaired by TDP-43^ΔNLS^ and FUS^ΔNLS^ in HEK293T cells. Utilizing Surface Sensing of Translation (SUnSET) (a non-radioactive method utilizing puromycin to tag newly synthesized proteins in live cells) [58], followed by western blotting (SUnSET-WB), we found that TDP-43^ΔNLS^ did suppress global translation in HEK293T cells **(**Fig. 4a, b). Similarly, both FUS^ΔNLS^ mutants also significantly suppressed global translation (Fig. 5a, b).

To validate these findings and visualize global translational changes in individual cells, SUnSET followed by immunocytochemistry (SUnSET-ICC) was performed in cells transfected with TDP-43^ΔNLS^ or FUS^ΔNLS^. As illustrated in Fig. 4c, TDP-43^ΔNLS^ aggregate-containing cells showed undetectable level of puromycin staining, in sharp contrast to neighbouring un-transfected (UT) HA-negative cells, consistent with strong translational suppression by TDP-43^ΔNLS^ aggregates. However, in cells where TDP-43^ΔNLS^ displayed a diffuse filamentary cytoplasmic expression pattern, translational level was comparable to neighbouring UT cells (Fig. 4c), suggesting that global translational suppression is predominantly caused by consolidated larger size TDP-43^ΔNLS^/RACK1 co-aggregates. Similar inhibition of puromycin incorporation was observed in FUS^ΔNLS^ aggregate-containing cells (Fig. 5c). Together, these results strongly suggest that co-aggregation of RACK1 with TDP-43^ΔNLS^ or FUS^ΔNLS^ significantly inhibits normal function of the translational machinery.

### TDP-43^ΔNLS^ and FUS^ΔNLS^/RACK1 co-aggregates sequester the polyribosome to form large aggregated complexes

**Fig. 6:**
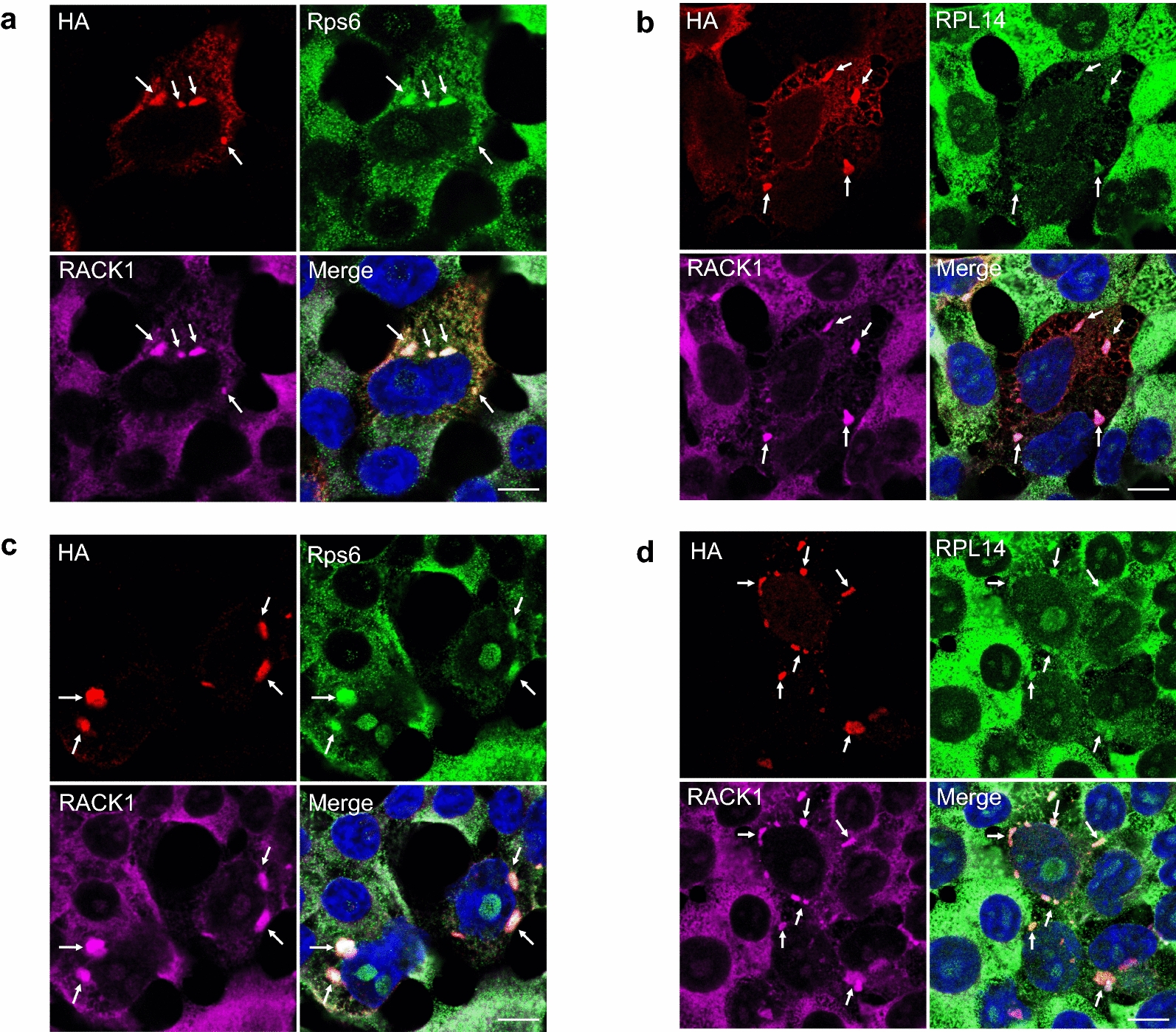
Immunocytochemical analysis shows co-aggregation of HA-tagged TDP-43^ΔNLS^ (**a**, **b**) or R495x-FUS (**c**, **d**) with RACK1 and eukaryotic 40S (Rps6) and 60S (RPL14) ribosome subunits (*arrows*). In neighbouring HA-negative UT cells, RACK1 is diffusely localized in the cytoplasm. Nuclei stained with DAPI in merged images. Scale bars: 10 μm

We next tested whether TDP-43^ΔNLS^ or FUS^ΔNLS^ inhibited global translation in our HEK293T cell system through sequestration of the polyribosome into RACK1 co-aggregates, as has been reported in SH-SY5Y cells [57]. Immunocytochemical analysis was performed to detect the association of TDP-43^ΔNLS^ or FUS^ΔNLS^/RACK1 aggregates with Rps6 and RPL14, two established markers for the eukaryotic small (40S) and large (60S) ribosomal subunits, respectively. As shown in Fig. 6, UT cells displayed the expected pattern of diffuse expression of Rps6 and RPL14 in the cytoplasm. In contrast, transfection with either TDP-43^ΔNLS^ or FUS^ΔNLS^ resulted in prominent co-localization with RACK1 and both Rps6 and RPL14. Together, these results demonstrate that TDP-43^ΔNLS^ and FUS^ΔNLS^ cytoplasmic aggregates may disrupt normal function of the translational machinery through aberrant sequestration of RACK1 and associated polyribosome into large aggregated complexes.

### RACK1 knockdown diminishes TDP-43^ΔNLS^ and FUS^ΔNLS^ cytoplasmic aggregation and leads to nuclear localization in a sub-population of cells

**Fig. 7:**
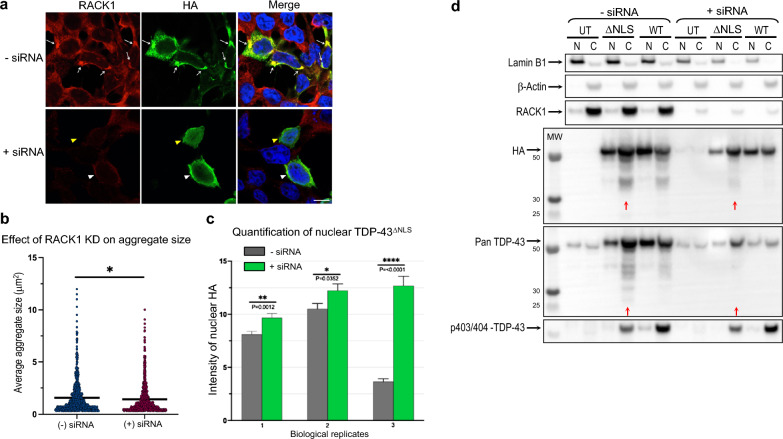
RACK1 knockdown not only reduces cytoplasmic aggregation but also increases nuclear localization of TDP-43^ΔNLS^ in HEK293T cells. **a** Representative images showing that in contrast to control cells where distinctive aggregates of HA-tagged TDP-43^ΔNLS^ are associated with RACK1 co-aggregation in the cytoplasm (*arrows*), RACK1 siRNA KD not only alleviates cytoplasmic aggregation (*white arrowheads*) but also leads to nuclear localization (*yellow arrowheads*) of TDP-43^ΔNLS^ in a sub-population of transfected cells. Nuclei stained with DAPI in merged images. Scale bar: 10 μm. **b** Quantification of the average aggregate size in each individual TDP-43^ΔNLS^ transfected cells shows a decrease following RACK1 KD. **c** Quantification of TDP-43^ΔNLS^ nuclear localization from 3 biological repeats shows an increase as a result of RACK1 KD. **d** Western blot analysis confirms the quality of nucleocytoplasmic fractionation using nuclear and cytosolic protein markers, lamin B1 and β-Actin, respectively (*top*). TDP-43^ΔNLS^ (HA) or total TDP-43 (pan TDP-43) from either fraction was normalized to β-actin or lamin B1. Quantification by densitometry shows that RACK1 KD decreases the cytoplasmic to nuclear ratio, fragmentation (*red arrows*, observed in 3 independent experiments), and phosphorylation of TDP-43^ΔNLS^ (quantitated in one experiment). *Statistics*: Student’s *t*-test unpaired two-tailed. Error bars: SEM

In light of the above results, we considered whether RACK1 knockdown (KD) would render a beneficial effect on TDP-43^ΔNLS^ or FUS^ΔNLS^ induced RACK1 aggregation. To this end, HEK293T cells were treated with human-specific anti-RACK1 siRNA prior to TDP-43^ΔNLS^ or FUS^ΔNLS^ transfections. As shown in Fig. 7a and b, KD of endogenous RACK1 significantly reduced the average aggregate size of TDP-43^ΔNLS^. A similar phenomenon was observed in FUS^ΔNLS^ transfected cells (Additional file 1: Fig. S5).

Surprisingly, RACK1 KD also resulted in partial redistribution of TDP-43^ΔNLS^ from being predominantly cytoplasmic to nuclear in a sub-population of transfected cells, despite its lack of nuclear localization signal (Figs. 4d, 7a and c). This observation was confirmed by biochemical analyses demonstrating a decreased cytoplasmic/nuclear ratio of TDP-43^ΔNLS^ (by ~ 42%) upon RACK1 KD (Fig. 7d). In addition, RACK1 KD led to a reduction of low molecular weight fragments and phosphorylated-TDP-43 in the cytoplasm by ~ 50% and ~ 21%, respectively (Fig. 7d), which are both hallmark biochemical features of TDP-43 pathology in affected CNS regions of ALS/FTLD patients [3, 11, 15, 44, 55, 63, 67]. Similar reversal from predominantly cytoplasmic to nuclear localization was observed with FUS^ΔNLS^ upon RACK1 KD (Fig. 5d, Additional file 1: Fig. S5a). It is notable that no detectable changes were observed with the morphology or viability of HEK293T cells upon RACK1 KD, and that endogenous TDP-43 and FUS retained their normal nuclear localization as in control RACK1 expressing cells (Additional file 1: Fig. S6).

### RACK1 KD restores global translational suppression by TDP-43^ΔNLS^ and FUS^ΔNLS^

In light of the above results showing that RACK1 KD reduced cytoplasmic aggregation and increased nuclear/cytoplasmic ratio of TDP-43^ΔNLS^ and FUS^ΔNLS^, we hypothesized that RACK1 KD may mitigate sequestration of polyribosomes into aggregates and thereby alleviate global translational suppression by both mutants. Utilizing SUnSET-WB and SUnSET-ICC, we showed that global translational suppression by either mutant was indeed significantly restored to levels comparable to those of controls by RACK1 KD (Figs. 4, 5).

### RACK1 KD inhibits TDP-43^ΔNLS^ intercellular transmission

**Fig. 8:**
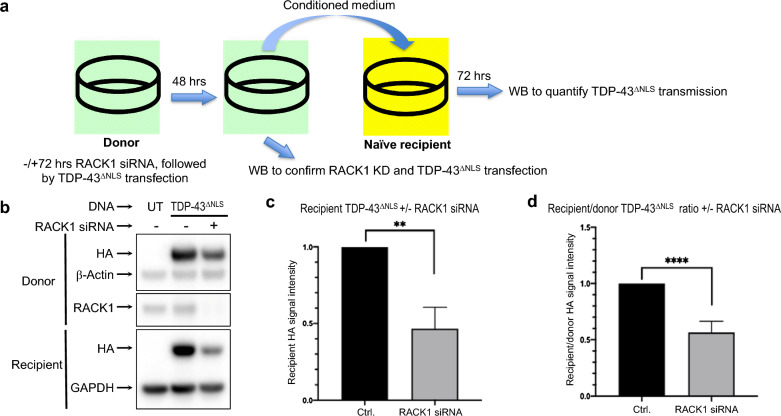
RACK1 knockdown inhibits intercellular transmission of TDP-43^ΔNLS^ in HEK293T cells. **a** Schematic illustration of the intercellular transmission procedure. Representative western blot (WB) **b** and densitometric quantification show reduced total HA-tagged TDP-43^ΔNLS^ transmitted to naïve recipient cells **c** as well as a reduction in the recipient/donor ratio of TDP-43^ΔNLS^ upon RACK1 KD. *Statistics*: Student’s *t*-test, unpaired two-tailed. **p < 0.005; ****p < 0.0001. n = 5

A body of evidence has suggested that misfolded/aggregated TDP-43 can be transmitted from cell to cell in a prion-like fashion and contributes to the spreading of neurodegeneration across CNS regions [9, 11, 18, 24, 28, 30, 53–55]. We therefore tested whether RACK1 KD might have an impact on cell-to-cell transmission of misfolded TDP-43^ΔNLS^. We employed a previously established procedure [23, 50], in which conditioned medium from donor cells expressing TDP-43^ΔNLS^ without or with RACK1 KD was added to naïve recipient cells for quantitation of transmitted TDP-43^ΔNLS^ (Fig. 8a). Compared to control donor cells, RACK1 KD reduced both total TDP-43^ΔNLS^ in recipient cells and the ratio of recipient/donor TDP-43^ΔNLS^ (Fig. 8b–d), indicating that RACK1 KD significantly inhibited the intercellular transmission of misfolded TDP-43^ΔNLS^.

### RACK1 KD alleviates retinal degeneration and delays the decline of motor function in human WT and mutant TDP-43 transgenic D. melanogaster

**Fig. 9:**
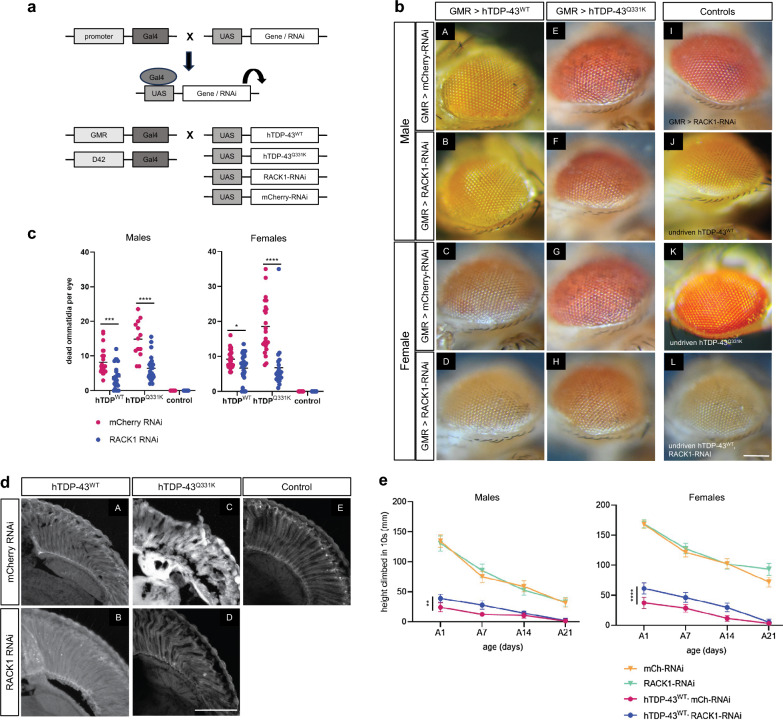
RACK1 knockdown significantly reduces hTDP-43-induced neuronal death and dysfunction in transgenic *D. melanogaster.* **a** Schematic illustration for the construction of transgenic *D. melanogaster* lines specifically targeting retinal (GMR-driven) or motor (D42-driven) neurons. **b** Representative images of fly eyes. Expression of hTDP-43^WT^ (A–D) or hTDP-43^Q331K^ (E–H) in retinal neurons cause neuronal death in both males and females, detected as darkened ommatidia. RACK1-RNAi reduces the appearance of dead ommatidia in all four conditions (B, D, F, H), compared to mCherry-RNAi control (A, C, E, G). Control flies with no dead ommatidia include those expressing RACK1-RNAi alone (I), and those harbouring unexpressed (undriven) transgenes (J–L). Scale bar: 250 mm. **c** Quantification of retinal neuronal death. Mann–Whitney test indicates a significant reduction in ommatidia death upon RACK1-RNAi KD in both males and females expressing either hTDP-43^WT^ or hTDP-43^Q331K^. 21–33 flies were scored per condition. *Statistics:* Mann–Whitney test, *p < 0.05, ***p < 0.001. ****p < 0.0001. **d** Histological analysis of retina from flies expressing hTDP-43^WT^ (A, B) or hTDP-43^Q331K^ (C, D) in conjunction with control mCherry**-**RNAi (A, C) or RACK1-RNAi (B, D) show preservation of retinal architecture by RACK1 KD. Scale bar: 50 mm. **e** Both male and female hTDP-43^WT^ transgenic flies show reduced climbing ability compared to mCherry-RNAi controls, which was significantly improved by RACK1-RNAi. *Statistics:* 2-way ANOVA. **p < 0.01, ****p < 0.0001. 60–100 flies were scored per condition. Error bars: SD

Finally, we sought to investigate whether the aforementioned beneficial effects of RACK1 KD on TDP-43^ΔNLS^ associated phenotypes in HEK293T cells could be translated into protective activity for neurons in vivo. To this end, we employed an established *D. melanogaster* UAS-Gal4 expression system to target expression of human wild-type TDP-43 and ALS-associated mutant TDP-43^Q331K^ (hTDP-43^WT^ and hTDP-43^Q331K^) [17] in retinal (driven by the GMR promotor) or motor (driven by the D42 promotor) neurons with or without knocking down of endogenous RACK1 by RACK1-RNAi co-expression (Fig. 9a), with mCherry-RNAi serving as a control.

Firstly, retinal degeneration was assessed for the loss of ommatidia and structural organization of the retina. As shown in Fig. 9b, compared to undriven controls (J–L), flies co-expressing mCherry-RNAi with hTDP-43^WT^ displayed mild neurodegeneration (A & C), consistent with a previous report [37], and the phenotype was more severe with expression of mutant hTDP-43^Q331K^ (E & G). Quantification of retinal degeneration (Fig. 9c) showed that in both male and female flies, hTDP-43^Q331K^ caused more neuronal cell death than hTDP-43^WT^ (Males: mean of 14.8 dead ommatidia out of ~ 700 per eye for hTDP-43^Q331K^ *vs*. 8.2 for hTDP-43^WT^; Females: 18.5 *vs.* 9.2). In contrast, flies co-expressing RACK1-RNAi with either hTDP-43^WT^ or hTDP-43^Q331K^ displayed significantly less degeneration in all four of these populations (B, D, F, H). Note that in several individuals expressing hTDP-43^WT^ with RACK1-RNAi, the number of dead ommatidia was reduced to zero. In contrast, in flies expressing either allele of hTDP-43 with control mCherry-RNAi, neuronal death was always observed (Fig. 9c). Additional evidence of neurodegeneration was observed in both hTDP-43^WT^ and hTDP-43^Q331K^ expressing flies, which displayed structural disorganization of the retina. These phenotypes were also significantly restored by RACK1-RNAi (Fig. 9d). Interestingly, flies expressing GMR-RACK1-RNAi alone (Fig. 9b, I) were comparable to undriven controls (Fig. 9b, J–L), suggesting that RACK1 may be dispensable in mature fly retinal neurons.

Secondly, the motor function of transgenic flies was assessed using a previously established climbing assay [59]. As shown in Fig. 9e, both male and female flies expressing hTDP-43^WT^ in motor neurons displayed reduced climbing ability compared to controls, which was significantly improved by RACK1-RNAi in both sexes, suggesting that knocking down of endogenous RACK1 delayed the decline of motor function caused by human TDP-43.

Together, these results demonstrate that knocking down of RACK1 significantly alleviated retinal degeneration and delayed motor neuron dysfunction caused by expression of hTDP-43^WT^ or hTDP-43^Q331K^ in *D. melanogaster*.

## Discussion

TDP-43 and FUS have drawn great interest in neuroscience since first identified as pathogenic proteins associated with ALS and FTLD. Because TDP-43 and FUS share many similarities in terms of their protein structure, nucleocytoplasmic distribution, physiological function, and histopathology in diseased human tissues, it is not surprising that a number of common cellular pathways have been identified to participate in the pathogenic activities of TDP-43 and FUS cytoplasmic aggregates. In the present study, we report our findings that support an additional shared pathway underlying the detrimental effects of TDP-43 and FUS aggregation in the cytoplasm, and explore a potential therapeutic avenue for the treatment of neurodegenerative diseases with TDP-43 and FUS proteinopathies.

### RACK1, a key player in pathogenic TDP-43 and FUS aggregation-induced global translational suppression

RACK1 can come in proximity and interact with TDP-43 and FUS under physiological conditions existing in normal cells, and in stress granules, which can transiently repress protein translation as a protective activity [68]. However, the interaction of RACK1 with protein aggregates in diseased cells may be pathologically significant and contribute to disruption of protein expression. In a *Drosophila* model of ALS, mutant TDP-43 was shown to repress translation of Futsch/Map1B, a key protein regulating axonal and dendritic development and microtubule organization at the neuromuscular junction [14]. In mouse primary neurons, mutant TDP-43, together with Fragile X Syndrome protein (FMRP), represses translation of neuronal proteins Rac1 and GluR1 [39]. In *Drosophila* and rat primary neurons, mutant TDP-43 induces a prolonged translational suppression by stimulating eIF2α phosphorylation [34]. Importantly, more recent studies using transfected human neuroblastoma cell lines or fibroblasts derived from ALS-FUS patients have provided strong evidence that TDP-43 and FUS cytoplasmic aggregates suppress global translation, and that mutant TDP-43 exerts this effect through direct interaction with the ribosome scaffold protein RACK1 [32, 57]. This work is consistent with our current findings that TDP-43^ΔNLS^ and FUS^ΔNLS^ mutants co-aggregate with RACK1 and suppress global translation in transfected HEK293T cells. Furthermore, we show that RACK1 KD not only reduced aggregation of TDP-43^ΔNLS^ and FUS^ΔNLS^, but also caused a shift of both mutants from being predominantly cytoplasmic to nuclear in a sub-population of transfected cells (discussed further below), accompanied by restoration of global translation, strongly suggesting that RACK1 is a key player in this aggregation/translational suppression process.

### RACK1—a scaffold linking pathogenic TDP-43 and FUS to ribosomes in formation of large aggregate complexes

To decipher the mechanism underlying the above observation, it is worthwhile to recapitulate the fundamental biological function of ribosomes. The ribosome is well appreciated as the central apparatus of protein synthesis, with the small (40S in eukaryotes) subunit initiating the mRNA to amino acid decoding/translational process while the large (60S in eukaryotes) subunit catalyzes the peptide transferase activity to generate nascent polypeptide chains. RACK1 is widely recognized to be an integral constituent of the 40S ribosomal subunit, which in turn is stably associated with the 60S subunit. Our confocal microscopic studies have demonstrated that TDP-43^ΔNLS^ and FUS^ΔNLS^ recruited both 40S and 60S subunits, in addition to RACK1, to the same aggregate complex in the cytoplasm (Fig. 6), resulting in global suppression of translation.

### Aggregate inhibition of global translation: size matters?

An interesting phenomenon observed from our SUnSET-ICC studies is that global translational suppression appears to be predominantly correlated with large distinctive aggregates of TDP-43^ΔNLS^ or FUS^ΔNLS^ but not the diffuse pool in the cytoplasm (Figs. 4c, 5c). This implies that global translation may not be significantly impaired unless the optical area of aggregated TDP-43 or FUS reaches a certain threshold. This is in line with previous findings using a motoneuron-like neuroblastoma cell line, NSC-34, which show that neuronal toxicity is attributed to the largest cytoplasmic aggregates of TDP-43 [6].

### Redistribution of NLS-deficient TDP-43 and FUS from predominantly cytoplasmic to nuclear expression upon RACK1 knockdown

Perhaps the most unexpected observation from the present study was that RACK1 KD changed the predominantly cytoplasmic mislocalization of TDP-43^ΔNLS^ and FUS^ΔNLS^ to a normal nuclear localization in a sub-population of transfected cells despite their lack of a functional NLS. One potential explanation is that the anti-aggregation activity of RACK1 KD reduced formation of compact large aggregates of TDP-43^ΔNLS^ and FUS^ΔNLS^ with the polyribosome, consequently allowing non-aggregated free molecules to diffuse from the cytoplasm to the nucleus. Given the current knowledge that cytosolic proteins are capable of crossing the nuclear pore complexes (NPC) through passive diffusion (as well as active transport), and that the likelihood of this process is not only inversely correlated with the molecular mass but also can be influenced by protein conformation [64], we speculate that TDP-43^ΔNLS^ and FUS^ΔNLS^ may have partly entered the nucleus through passive diffusion, as opposed to the classical NLS-dependent active nuclear import pathway for physiological TDP-43 and FUS [4]. Moreover, the cutoff molecular mass for passive diffusion across NPC can be up to 90-110kD [69], which encompasses the molecular weights of monomer/dimer TDP-43 and monomer FUS.

### RACK1 knockdown rescues TDP-43-associated neurodegeneration in vivo

A most important question arising from the present study is whether the healthy nuclear TDP-43 and FUS are capable of carrying out their physiological functions in cells in which RACK1 KD attenuates the toxic effects of TDP-43^ΔNLS^ or FUS^ΔNLS^. Healthy TDP-43 and FUS, as nuclear ribonucleoproteins, are well-known for their role in alternative splicing regulation of specific mRNA targets as well as mRNA transport and stability [2, 5, 16, 22, 26, 27, 52, 65]. In the present study, we directly tested RACK1 KD strategy in *Drosophila* models transgenic for human WT or mutant TDP-43 exclusively in retinal or motor neurons in vivo. We found that RACK1 KD significantly alleviated retinal neurodegeneration and delayed locomotion deficit of TDP-43 transgenic flies, indicating that RACK1 KD allows normal healthy fly TDP-43 to perform its physiological functions. Moreover, flies with RACK1 KD alone were not only viable, but also exhibited retinal health comparable to control flies, suggesting that physiological level of RACK1 may not be required for the survival and function of differentiated neurons in flies (Fig. 9). This is in agreement with previous findings that knocking down of RACK1 does not affect mature cell viability and proliferation in *Drosophila* [40], despite lethality when expressed in early development, particularly oogenesis [31]. Similar strategies can be employed in future studies to characterize the effect of RACK1 KD on transgenic flies expressing human FUS mutations [35, 62].

Overall, our results reveal a novel shared mechanism of pathogenesis for misfolded aggregates of TDP-43 and FUS mediated by interference with protein translation in a RACK1-dependent manner and provide proof-of-concept evidence for targeting RACK1 as a therapeutic approach for TDP-43 or FUS proteinopathy associated with ALS and FTLD.

## Materials and methods

### Cell culture, plasmids, and transfection

#### Cell culture

Human embryonic kidney 293T (HEK293T) cell line was purchased from American Type Culture Collection (ATCC, Rockville, MD, USA), and maintained in Dulbecco's Modified Eagle Medium (DMEM) supplemented with 10% fetal bovine serum (FBS), GlutaMax™ (2 mM) and antibiotics (50 U/ml penicillin and 50 μg/ml streptomycin) at 37 °C in 5% CO_2_.

#### Plasmids

HA-tagged WT TDP-43, TDP-43^ΔNLS^ (K82A/R83A/K84A), WT FUS, R495x-FUS, and P525L-FUS FUS (collectively designated FUS^ΔNLS^) were generated as previously described [51]. His-Myc-tagged R38D/K40E-RACK1 (DE-RACK1) was a kind gift from Marcello Ceci lab (Tuscia University, Italy). Flag-tagged human long variant (Lv) isoform of DISC1, 10W/S-DISC1, was constructed by GenScript Biotech (Piscataway, NJ, USA).

#### Transfection

cDNA plasmids were delivered into HEK293T cells using Lipofectamine LTX reagent (ThermoFisher Scientific, Waltham, MA, USA) following the manufacturer’s instruction, and cells were analyzed 48 h post-transfection. RACK1 KD was achieved by transfection of a pool of 3 target-specific siRNA plasmids against human RACK1 (Santa Cruz Biotechnology, Dallas, TX, USA, sc-36354) using Lipofectamine RNAiMAX reagent (ThermoFisher) according to the manufacturer’s instruction, and incubated for 72 h prior to cDNA plasmid transfection where indicated.

### SUnSET

To monitor global translation, cells were treated with 5 µg/ml of puromycin (ThermoFisher) for 10 min at 37 °C in conditioned media, and lysed in 2% SDS for total protein extraction, electrophoresis, and western blotting quantification or fixed in 4% paraformaldehyde (PFA) for immunocytochemical and microscopic analysis.

### Immunocytochemistry

HEK293T cells were washed twice with Phosphate Buffered Saline (PBS) and fixed in 4% PFA for 15 min at room temperature (RT), followed by wash with 20 mM glycine for 10 min at RT with constant rocking to quench residual PFA. Cells were then incubated with blocking buffer containing PBS, 1% Bovine Serum Albumin (BSA), 10% normal goat serum, and 0.1% Triton-X-100 for 30 min at RT. The following primary antibodies were incubated for 1 h at RT or overnight at 4 °C: rabbit polyclonal anti-HA (Abcam, Cambridge, UK, ab9110, 1:1,000), chicken polyclonal anti-HA (Abcam, ab9111, 1:10,000), rabbit polyclonal anti-c-Myc (Abcam, ab9106, 1:1,000), mouse monoclonal anti-RACK1 (BD Biosciences, San Jose, CA, USA, 610178, 1:500), rabbit monoclonal RACK1^mis^ (ProMIS Neurosciences, see below, 1 μg/ml), rabbit monoclonal anti-RpS6 (Cell Signaling, Danvers, MA, USA, 5G10, 1:100), rabbit polyclonal anti-RPL14 (Bethyl Laboratories, Montgomery, TX, USA, A305-052, 1:1,000), mouse monoclonal anti-puromycin (ThermoFisher, clone 12D10, 1:1,000), rabbit monoclonal anti-Flag (Cell Signaling, 2368S, 1:2,000). Cells were then washed with PBS/0.1%Triton-X-100 three times for 10 min with constant rocking, followed by incubation with Alexa Fluor® goat anti-rabbit, -mouse, or -chicken secondary antibody (ThermoFisher, 1:1,000) for 30 min at RT in the dark. Cells were then washed with PBS/0.1%Triton-X-100 three times for 10 min, dipped in 5% PBS, and mounted with ProLong Gold Anti-fade Reagent with DAPI (ThermoFisher, P36931). Cells were analyzed by confocal microscopy (Leica TCS SP8 MP, Wetzlar, Germany).

The RACK1^mis^ monoclonal antibody was generated by ProMIS Neurosciences using a proprietary computational modeling method, “Collective Coordinates” [48], to identify regions of RACK1 thermodynamically likely to be exposed on misfolded/unfolded RACK1 but not on the properly folded form of the protein. Rabbits were immunized with the predicted epitopes to generate monoclonal antibodies that were screened by ICC for recognition of RACK1 present in cytoplasmic aggregates of HEK293T cells transfected with TDP-43^ΔNLS^ or R495x-FUS, and without detectable staining of diffuse, physiological RACK1 present in the cytoplasm. The staining properties of the RACK1^mis^ antibody are shown in Additional file 1: Fig. S7.

### Immunoblotting

Cells were washed twice with ice-cold PBS and lysed in 2% SDS, followed by sonication at 30% power for 15 s to extract total protein. Protein content was determined by BCA assay (ThermoFisher). 10 µg of protein was separated on 4–12% NuPAGE Bis–Tris SDS-PAGE (ThermoFisher), transferred onto a PVDF membrane, and blocked in Tris buffered saline (TBS) containing 5% skim milk and 0.1% Tween-20 for 1 h at RT. The following primary antibodies were incubated overnight at 4 °C: rabbit anti-HA (Abcam, ab9110, 1:1,000), mouse anti-RACK1 (BD Biosciences, 610178, 1:2,000), mouse anti-puromycin (ThermoFisher, clone 12D10, 1:10,000), mouse anti-α-tubulin (ProteinTech, Rosemont, IL, USA, 66031-1-Ig, 1:20,000), mouse anti-β-actin (Applied Biological Materials, Richmond, BC, Canada, G043, 1:1,000), rabbit anti-lamin B1 (abcam, ab16048, 1:1,000), mouse anti-TDP-43 (ProteinTech, 60019-2-Ig, 1:5,000), rabbit anti-phospho TDP-43 pS403/404 (Cosmo Bio, Carlsbad, CA, USA, TIP-PTD-P05, 1:3,000), mouse anti-GAPDH (ThermoFisher, AM4300, 1:100,000). Membranes were washed with TBS/0.1%Tween (TBST) three times for 10 min at RT with constant rocking, followed by horseradish peroxidase (HRP)-conjugated goat anti-mouse IgG (Sigma, St. Louis, MI, USA, AP181P, 1:5,000) or donkey anti-rabbit secondary IgG secondary antibody (Sigma, AP182P, 1:5,000) incubation for 30 min at RT. Membranes were then washed with TBST three times for 10 min, and developed with SuperSignal™ West Femto Maximum Sensitivity Substrate (ThermoFisher).

### Immunohistochemistry

For fluorescence-based IHC, paraffin-embedded spinal cord sections (6 μm) from healthy control, fALS-C9orf72, fALS-FUS-R521C, or sALS (kindly gifted by Drs. Ian Mackenzie and Janice Robertson) were de-paraffinized for 20 min at 55 °C, followed by rehydration in xylene, xylene/ethanol (1:1), 100%, 95%, 75%, 50% of ethanol, and TBS. The sections were then exposed to sub-boiling antigen retrieval buffer containing 20 mM sodium citrate pH 6.0 for 10 min, cooled at RT for 30 min, and incubated in blocking buffer containing TBS, 10% normal goat serum (NGS), 3% BSA, and 0.3% Triton-X-100 for 2 h, prior to incubation with primary antibodies diluted in background-reducing antibody diluent (Agilent, Santa Clara, CA, USA) overnight at 4 °C in a humidified chamber. The following antibodies were used: mouse anti-RACK1 (BD Biosciences, 610178, 1:100), rabbit anti-FUS (Sigma, HPA008784, 1:1,000), rabbit monoclonal RACK1^mis^ (ProMIS Neurosciences, 1 μg/ml). Sections were then washed in TBS/0.3% Triton-X-100 (TBS-T) three times for 5 min with constant rocking, followed by incubation with Alexa Fluor^®^ goat anti-rabbit, or -mouse IgG secondary antibody (ThermoFisher, 1:500) for 45 min at RT in the dark, and washed in TBS/0.3% Triton-X-100 3 X 5 min. Auto-fluorescence was then quenched by 0.1% Sudan Black B in 70% ethanol. The sections were finally mounted with ProLong Gold Anti-fade Reagent with DAPI (ThermoFisher), and analyzed by confocal microscopy (Leica TCS SP8 MP, Wetzlar, Germany).

For chromogenic IHC, fresh frozen cryo-sections (25 μm) of AD frontal cortex brains and sALS cervical spinal cords (kindly gifted by Dr. Ian Mackenzie) were fixed in 10% NBF for 5 min, washed in TBS three times for 10 min, followed by incubation with 0.3% H_2_O_2_ in methanol for 30 min at RT to quench endogenous peroxidase. Sections were washed in TBS-T, three times for 5 min, and incubated in blocking buffer for 2 h at RT. Endogenous biotin was then blocked by incubation with Avidin (Vector Laboratories, Newark, CA, USA) for 15 min at RT and two 5 min washes in TBS-T prior to incubation with primary antibodies diluted in background reducing antibody diluent (Agilent) overnight at 4 °C. The following antibodies were used: mouse anti-RACK1 (BD Biosciences, 1:100), rabbit RACK^mis^ (ProMIS Neurosciences, 1 μg/ml). Sections were then washed in TBS-T three times for 10 min, followed by incubation with biotin F(ab)’2 fragment goat anti-mouse or -rabbit IgG secondary antibody (ThermoFisher) for 1 h at RT, washed in TBS-T, rinsed briefly in TBS, prior to amplification in Avidin–Biotin Complex (Vector) for 45 min at RT. VIP HRP substrate (Vector) was then applied to the sections for 2 min, followed by nuclear counterstaining in Methyl Green (Vector) for 2 min. Sections were cleared by dehydration in ethanol and incubation in Citrasolv (Decon Labs, PA, USA) twice for 10 min. Finally, sections were mounted onto microscopic slides in Permount mounting medium (ThermoFisher).

### Nucleocytoplasmic fractionation

Cells were washed twice in ice-cold PBS, followed by centrifugation at 3000 RPM for 5 min at 4 °C. Cell pellets were resuspended in cytoplasm extraction buffer containing 10 mM HEPES (pH 7.9), 10 mM KCl, 1 mM EDTA (pH 8), 1 mM EGTA (pH 8), 0.1% NP-40, 1 mM PMSF, and protease inhibitor cocktail (ThermoFisher) and homogenized 10 times using a 25-gauge needle, followed by rotation for 15 min at 4 °C and centrifugation at 3000 RPM for 5 min at 4 °C. Supernatant was collected as the cytosolic fraction. Pellets were washed three times in cytoplasm extraction buffer, then resuspended in nuclear extraction buffer containing 20 mM HEPES (pH 7.9), 420 mM NaCl, 1 mM EDTA (pH 8), 1 mM EGTA (pH 8), 0.5% NP-40, 0.1% SDS, 10% glycerol, 1 mM PMSF, and protease inhibitor cocktail (ThermoFisher), and homogenized 10 times using a 25-gauge needle. The mixture was sonicated for 3 s at 20% power, followed by centrifugation at 14,000 RPM for 30 min at 4 °C. Supernatant was collected as the nuclear fraction.

### Intercellular transmission

Intercellular transmission assay was performed following a previously established procedure and is illustrated in Fig. 8a. Briefly, donor cells were transfected with RACK1 siRNA or left un-transfected as control for 72 h, followed by TDP-43^ΔNLS^ cDNA plasmid transfection for an additional 48 h. Donor cells were then collected and subjected to western blot analysis for confirmation of RACK1 siRNA activity and TDP-43^ΔNLS^ expression. The conditioned medium from donor cells was centrifuged at 1000 RPM for 5 min at RT to remove cell debris. Supernatant was then added to naïve recipient cells and incubated for 72 h, followed by western blot analysis for quantitation of transmitted TDP-43^ΔNLS^.

### Drosophila melanogaster in vivo studies

#### Source of *D. melanogaster*

The following publicly available *D. melanogaster* lines were obtained from Bloomington Drosophila Stock Center (BDSC, Bloomington, IN, USA): GMR-Gal4 (BDSC #9146), D42-Gal4 (BDSC #7009), UAS-hTDP-43^WT^ (BDSC #79587) [17], UAS-hTDP-43^Q331K^ (BDSC #79590) [17], UAS-RACK1-RNAi TRiP.HMS01171 (BDSC #34692), UAS-mCherry-RNAi (BDSC #35785).

#### External eye imaging

For eye analysis, flies were maintained at 25 °C. Photographs of fly external eyes were obtained using a Zeiss Stemi SV 11 dissection microscope at 6× magnification equipped with an Olympus OM-D E-M1 digital camera controlled by Olympus Image Share app. Dead ommatidia were counted visually using a dissecting microscope, with the experimenter blinded to condition. 21–33 flies were scored per condition. Each data point is the mean of left and right eye for one individual.

#### Preparation of fly eyes for histology

Flies were anaesthetized with CO_2_ and decapitated. Heads were fixed in 4% PFA 0.2% Triton-x in PBS for 2 h at RT, and then incubated in 30% sucrose in PBS for 2 h at 4 °C for cryopreservation. Heads were embedded in OCT (Tissue-Tek) in rubber molds and frozen at − 80 °C. Tissue was cryo-sectioned at − 20 C into 10 µm slices, mounted onto slides, and cover-slipped using Vectashield (Vector Laboratories, Newark, CA, USA). No counter-stain was needed as retinal pigments are highly autofluorescent between approximately 470–600 nm excitation.

#### Imaging and analysis of retina

Retinal sections were viewed on an Olympus FLUOVIEW FV1000 Confocal Laser Scanning Biological Microscope with Fluoview software. Single plane images were captured using 488 nm excitation with a 20 × objective. Image files were code-named in order to blind the experimenter to genotype.

#### Climbing assay

The locomotion of flies was performed as previously described [59]. Cohorts of 15 adult flies, which had been allowed to mate for 1–2 days after eclosion, were separated by sex and housed in their testing cohorts under controlled conditions of 23 °C, 50–60% humidity, and 12-h on/off light cycle. Each week, 6–9 h into the 12-h light cycle of the flies (coinciding with their most stable activity levels), the climbing assays were conducted. Lighting and environmental conditions were carefully controlled in the testing area. Flies were anaesthetized briefly with CO_2_ and placed in a 100 ml measuring cylinder. They were given 15 min to recover from anesthesia and to acclimatize to the cylinder. The measuring cylinder was tapped 3 times upon a mouse pad to send the flies to the bottom, and a still photograph was taken 10 s later using an iPhone. Flies were then placed onto fresh food until the following week. From the photographs, the location of each fly in the graduated cylinder and distance climbed was noted. > 60 flies per genotype were measured, and the results analyzed by two-way ANOVA.

### Statistics

Statistical analysis was performed using GraphPad Prism (GraphPad Software, San Diego CA, USA). ANOVA, Mann–Whitney, or Student’s *t*-test was performed where appropriate as indicated to determine the significant differences. * *p* ≤ 0.05; ** *p* ≤ 0.01; *** *p* ≤ 0.001; **** *p* ≤ 0.0001.

### Supplementary Information

**Additional file 1:** **Fig. S1**: RACK1 and TDP-43 co-aggregate in ALS spinal cord motor neurons. **Fig. S2**: Endogenous TDP-43 and FUS expressions are not affected by mutant RACK1. **Fig. S3**: RACK1 does not co-aggregate with mutant DISC1. **Fig. S4**: RACK1 is misfolded in ALS spinal cord and FTLD-TDP brain tissues. **Fig. S5**: RACK1 knockdown reduces cytoplasmic aggregation of P525L-FUS in HEK293T cells. **Fig. S6**: Endogenous TDP-43 and FUS expressions are not affected by RACK1 knockdown. **Fig. S7**: Validation of the RACK1^mis^ antibody.

## Data Availability

The datasets generated in the current study are available from the corresponding author on reasonable request.
