# Supplementary material for: Targeting RACK1 to alleviate TDP-43 and FUS proteinopathy-mediated suppression of protein translation and neurodegeneration
Source: Acta Neuropathol Commun. 2023 Dec 18;11:200. doi: 10.1186/s40478-023-01705-8 (PMC10726565; doi:10.1186/s40478-023-01705-8)
Supplement: Supplementary file 1 — Additional file 1: Fig. S1: RACK1 and TDP-43 co-aggregate in ALS spinal cord motor neurons. Fig. S2: Endogenous TDP-43 and FUS expressions are not affected by mutant RACK1. Fig. S3: RACK1 does not co-aggregate with mutant DISC1. Fig. S4: RACK1 is misfolded in ALS spinal cord and FTLD-TDP brain tissues. Fig. S5: RACK1 knockdown reduces cytoplasmic aggregation of P525L-FUS in HEK293T cells. Fig. S6: Endogenous TDP-43 and FUS expressions are not affected by RACK1 knockdown. Fig. S7: Validation of the RACK1mis antibody. [file 40478_2023_1705_MOESM1_ESM.pdf]

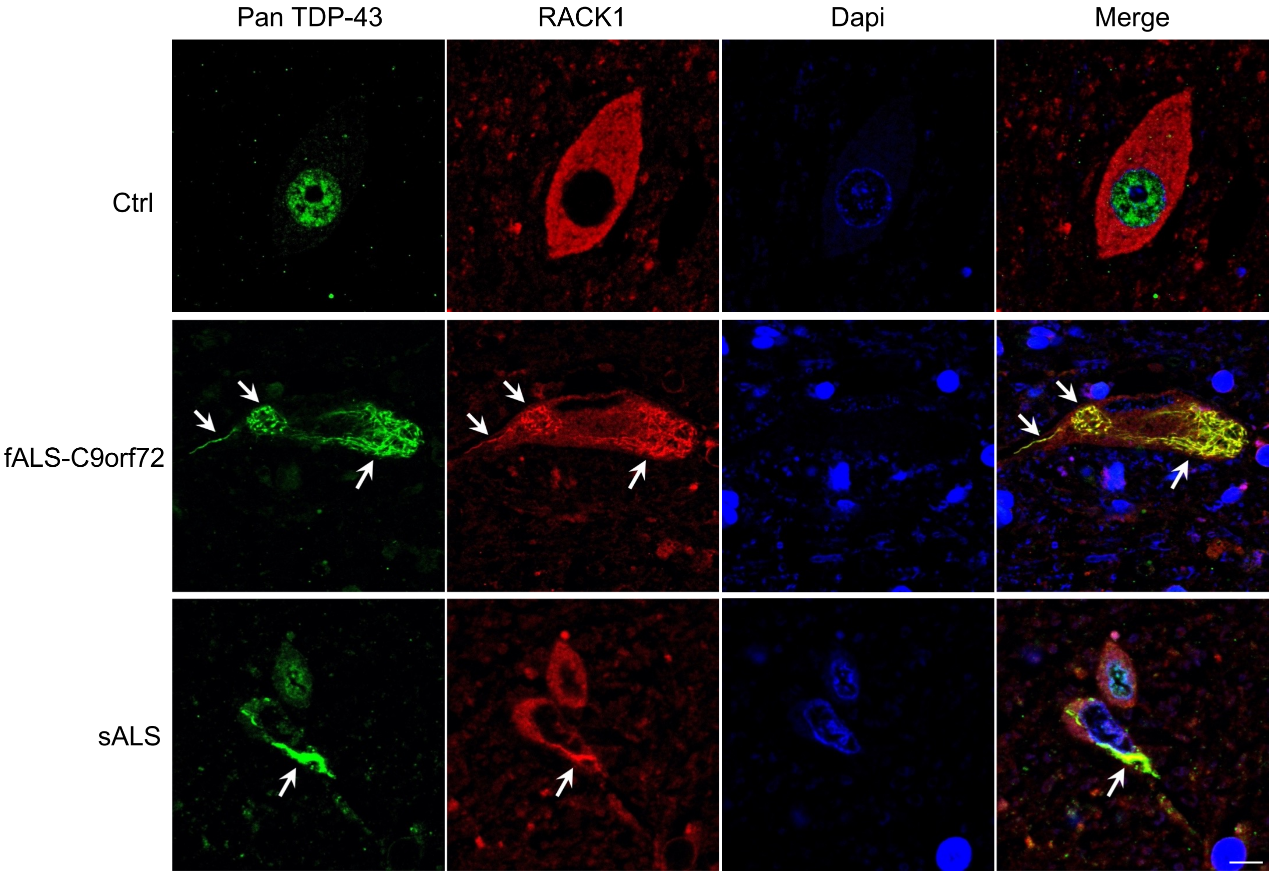

Fig. S1 RACK1 and TDP-43 co-aggregate in ALS spinal cord motor neurons. Representative images of immunohistochemical analysis showing co-aggregation of RACK1 with TDP-43 in the cytoplasm (*arrows*) in spinal cord sections of C9orf72-linked familial ALS (fALS) and sporadic ALS ((sALS) cases, in contrast to normal nuclear expression of TDP-43 in control spinal cords (Ctrl). Scale bar: 10 μm.

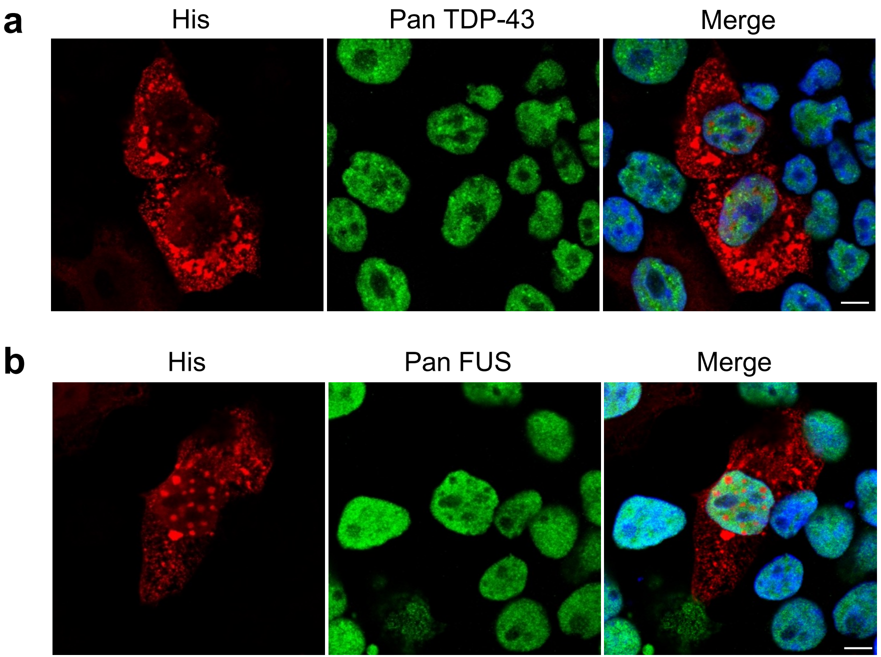

Fig. S2 Endogenous TDP-43 and FUS expressions are not affected by mutant RACK1. Immunocytochemical analysis shows His-tagged DE-RACK1-containing aggregates do not alter the normal nuclear expressions of endogenous TDP-43 **(a)** and FUS **(b)** in HEK293T cells. Scale bars: 20 μm.

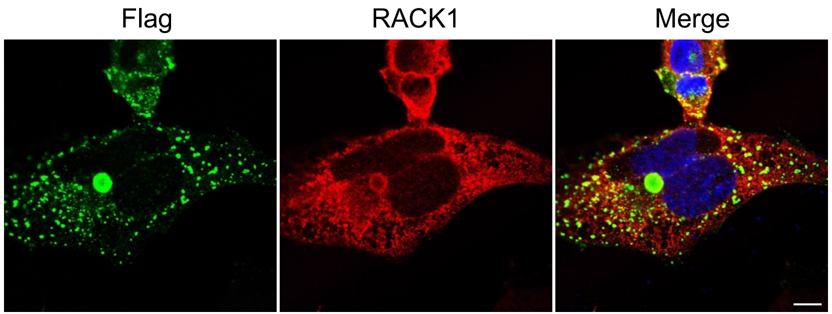

Fig. S3 RACK1 does not co-aggregate with mutant DISC1. Immunocytochemical analysis shows minimal association of Flag-tagged 10W/S-DISC1 mutant aggregates with endogenous RACK1 in HEK293T cells. Scale bar: 20 μm.

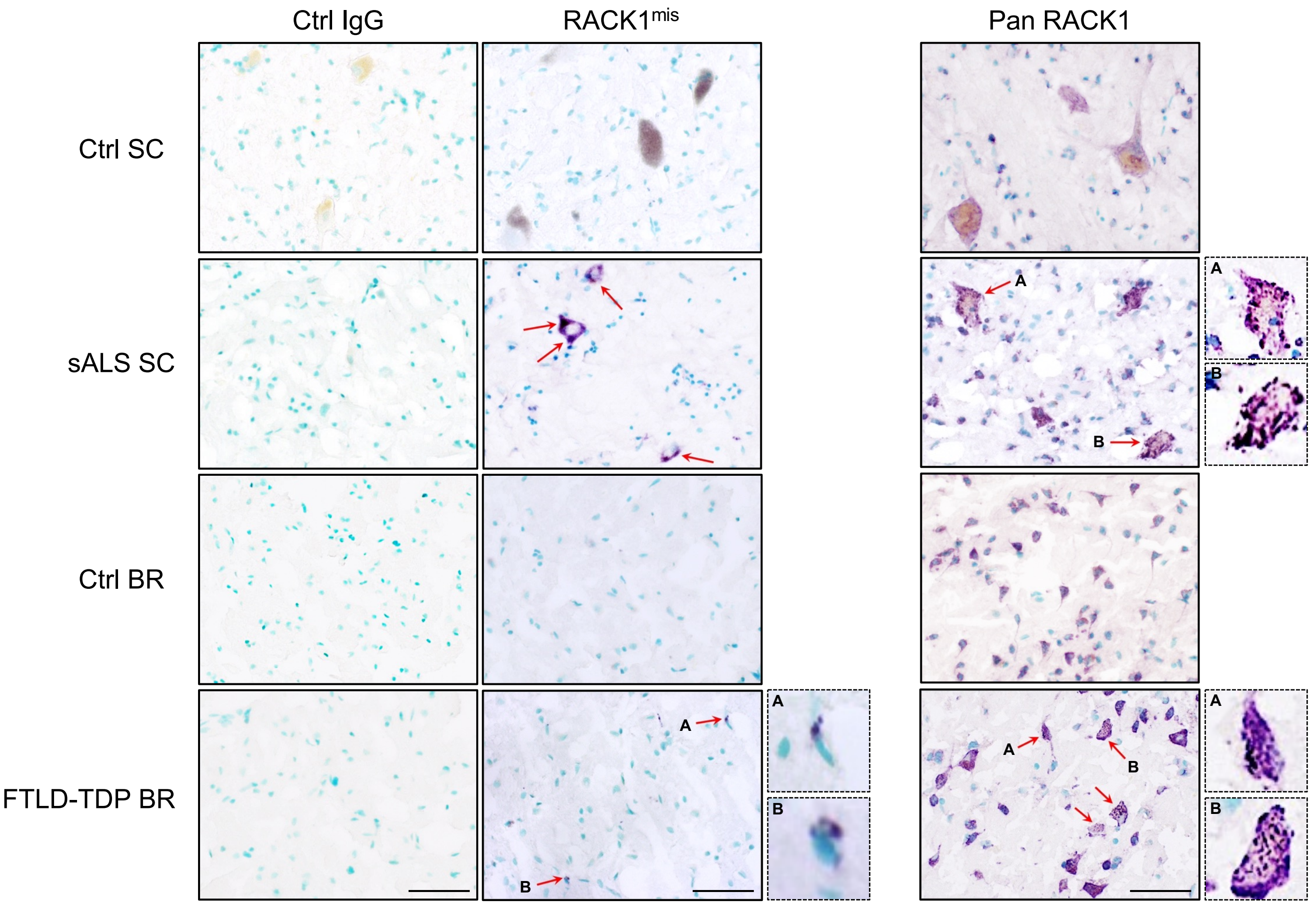

Fig. S4 RACK1 is misfolded in ALS spinal cord and FTLD-TDP brain tissues. Immunohistochemical analysis demonstrates the immunoreactivity of RACK1 misfolding-specific antibody “RACK1^{mis}” with cytoplasmic aggregates in sporadic ALS spinal cord (sALS, SC) and FTLD-TDP brain (BR) (*arrows*) with no reactivity in corresponding control (Ctrl) tissues. Pan RACK1 antibody reacts with both aggregated and normal RACK1 in all tissues. Scale bars: 50 μm.

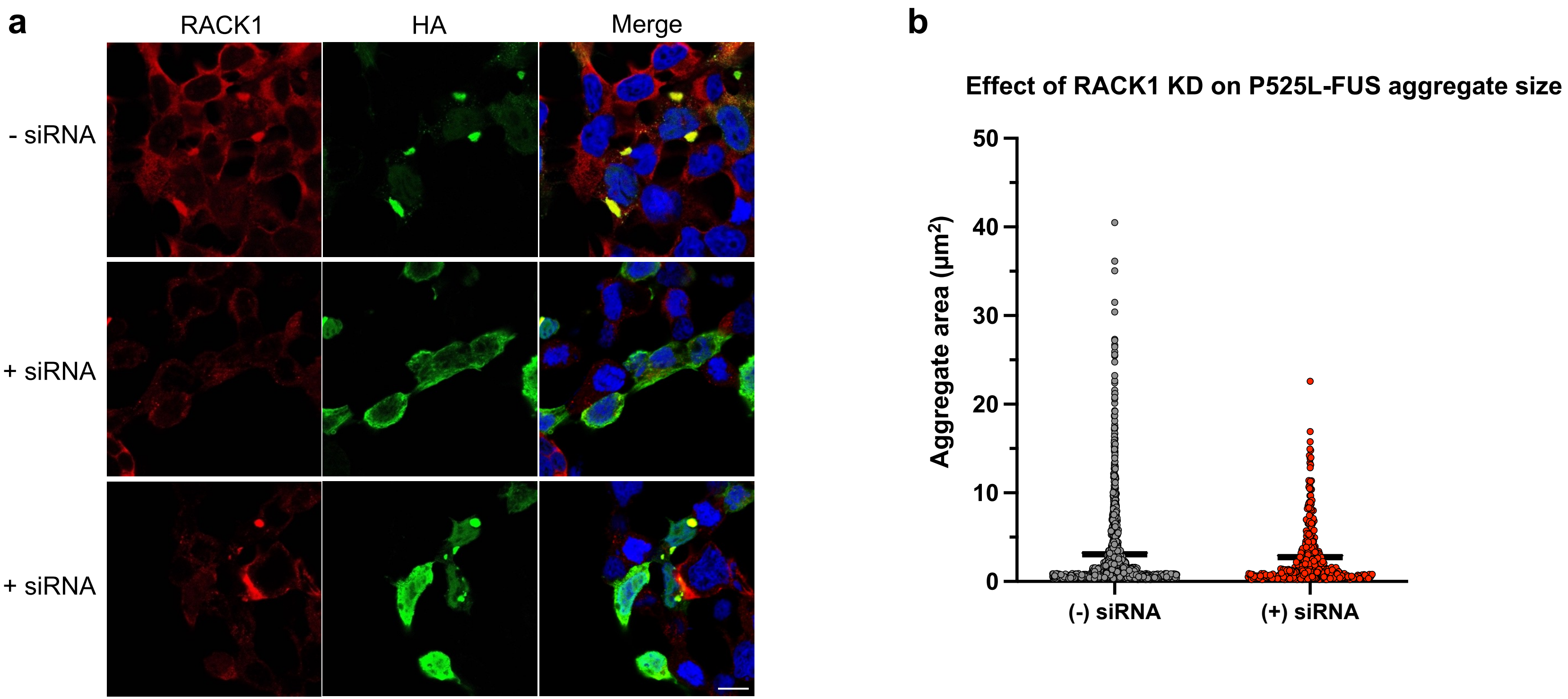

Fig. S5 RACK1 knockdown reduces cytoplasmic aggregation of P525L-FUS in HEK293T cells. (a) Representative images showing that in contrast to control cells where distinctive aggregates of HA-tagged P525L-FUS induce RACK1 co-aggregation in the cytoplasm (*top row*, -siRNA), RACK1 KD not only ameliorates cytoplasmic aggregation (*middle row*) but also leads to nuclear localization of P525L-FUS in a sub-population of transfected cells (*bottom row*). **(b)** Quantification shows a trend for RACK1 to reduce the average size of P525L-FUS aggregates in each individual transfected cells, although it does not reach statistical significance (p=0.28). *Statistics*: Student's t-test unpaired two-tailed. Error bars: SEM. Scale bar: 10 μm.

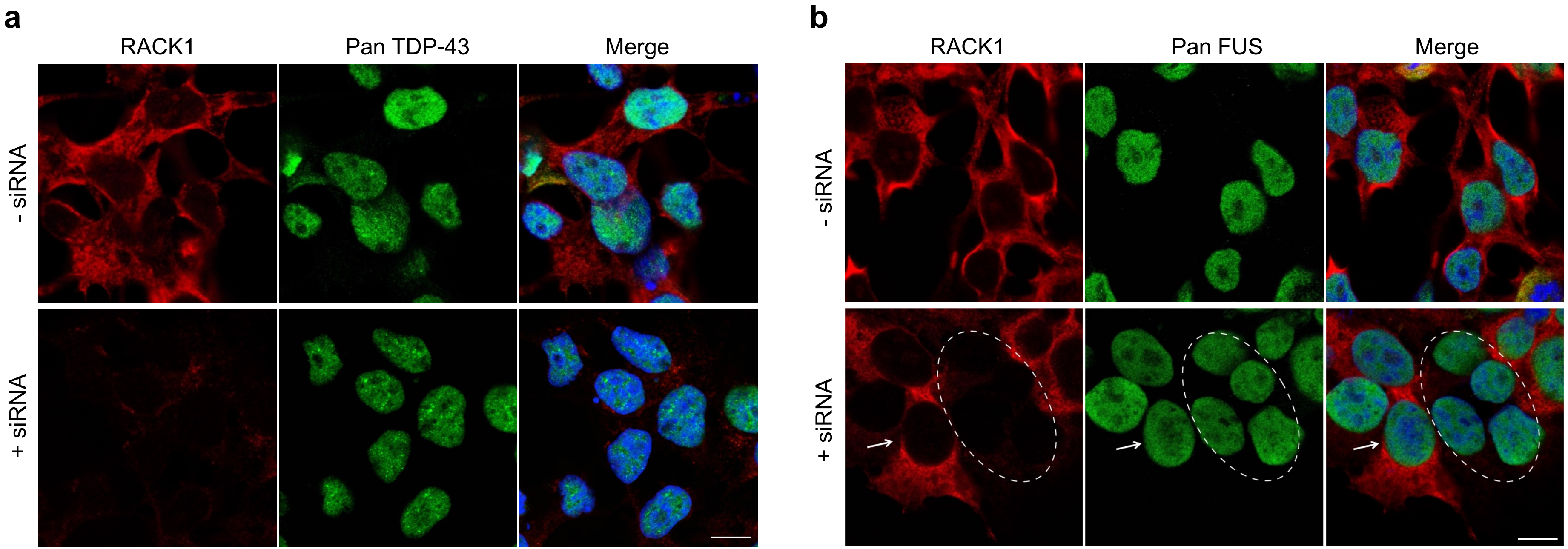

Fig. S6 Endogenous TDP-43 and FUS expressions are not affected by RACK1 knockdown. Endogenous TDP-43 (Pan TDP-43) **(a)** and FUS (Pan FUS) **(b)** retain normal nuclear expression upon RACK1 knockdown, similar to control RACK1 expressing cells. In *panel b*, *dashed circles*: cells with RACK1 KD; *arrows*: RACK1 expressing cells for direct comparison within the same field. Scale bars: 10 μm.

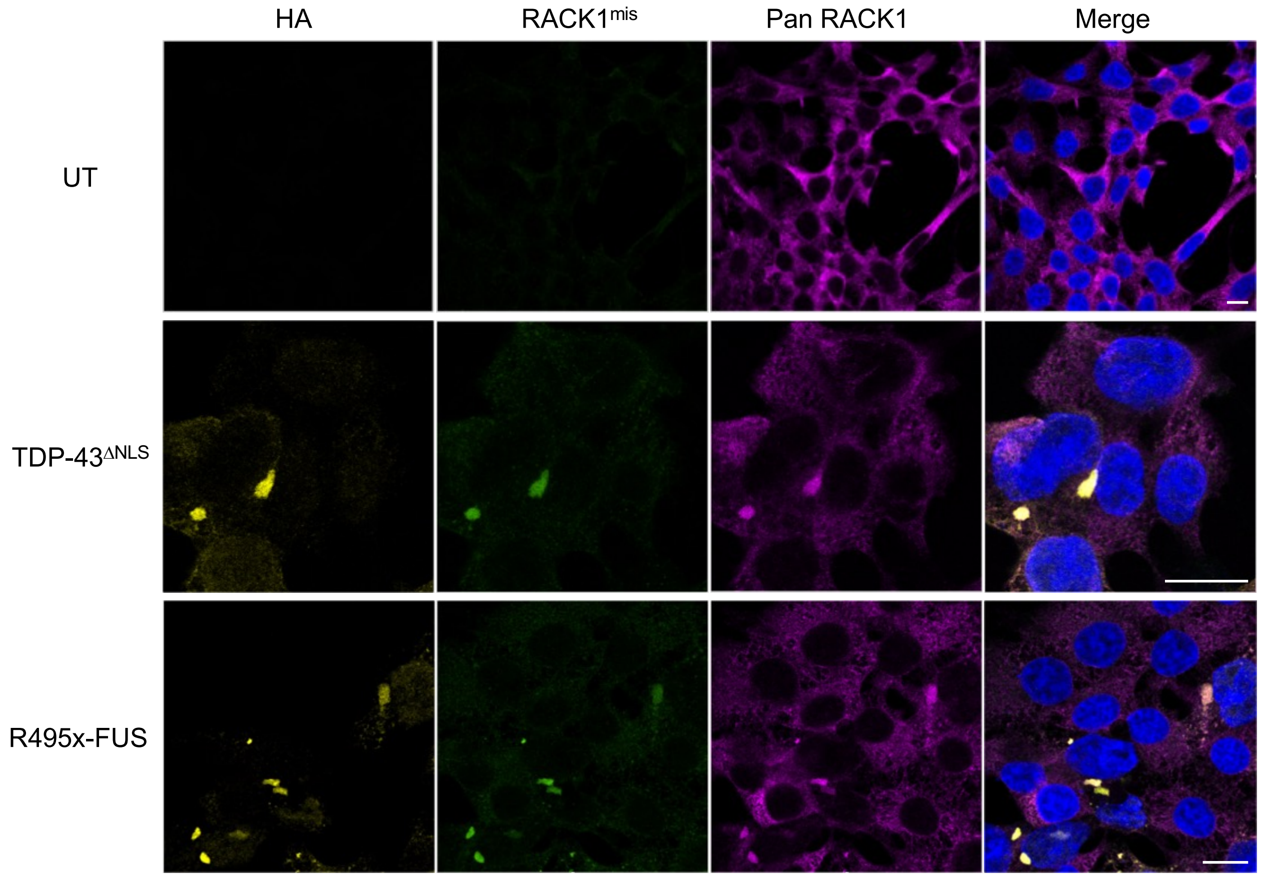

Fig. S7 Validation of the RACK1^{mis} antibody. Immunocytochemical analysis demonstrates the selective binding pattern of RACK1 misfolding specific antibody “RACK1^{mis}”. RACK1^{mis} reacts with RACK1 in cytoplasmic aggregates of HA-tagged TDP-43$^{\Delta NLS}$ (*middle row*) or R495x-FUS (*bottom row*) transfected HEK293T cells but not with diffuse, non-aggregated RACK1 in the cytoplasm (stained by a Pan RACK1 antibody). RACK1^{mis} shows no reactivity with endogenous, physiological RACK1 in un-transfected (UT, *top row*) cells. Scale bars: 10 μm.
